# Urban–rural differences in determinants of mental health among primary healthcare workers in China

**DOI:** 10.1017/S2045796025100425

**Published:** 2026-01-07

**Authors:** Jiaoling Huang, Yuqi Yang, Yijing Chu, Ping Zhu, Hong Liang, Jie Gu, Yan Li

**Affiliations:** 1 School of Public Health, Shanghai Jiao Tong University School of Medicine, Shanghai, China; 2 Shanghai Institute of Infectious Disease and Biosecurity, Fudan University, Shanghai, China; 3 School of Social Development and Public Policy, Fudan University, Shanghai, China; 4 Department of General Practition, Zhongshan Hospital Fudan University, Shanghai, China; 5 International Medical Center, Zhongshan Hospital Fudan University, Shanghai, China; 6 Department of Population Health Science and Policy, Icahn School of Medicine at Mount Sinai, New York, NY, USA

**Keywords:** anxiety, Bayesian Additive Regression Tree, China, depression, mental health, primary care, primary healthcare workers, risk factors, rural, urban

## Abstract

**Aims:**

The mental health risk factors for primary healthcare workers (PHWs) following the Coronavirus Disease 2019 pandemic and the differences by urbanicity remain unclear. In this study, we aimed to identify key factors of anxiety and depression among PHWs in urban and rural settings in China.

**Methods:**

This cross-sectional study was conducted in all 31 provinces in mainland China, between 1 May and 31 October 2022. A total of 3,769 PHWs, including family physicians, nurses, public health professionals, pharmacists, and other medical staff, were recruited from 44 urban community health service centers and 27 rural township hospitals. The Bayesian Additive Regression Tree model was employed to identify risk factors of anxiety and depression.

**Results:**

Among 3,769 PHWs, 1,006 (26.7%) worked in urban areas and 2,763 (73.3%) in rural areas. Occupational satisfaction significantly influenced anxiety in both urban and rural practitioners. For urban PHWs, living with family (odds ratio (OR): 0.42, 95% confidence interval (CI): 0.28–0.62) and self-rated health (fair: OR: 0.31, 95% CI: 0.23–0.42; good: OR: 0.13, 95% CI: 0.09–0.20) were key factors of anxiety. For rural PHWs, after-work exercise (rarely: OR: 0.28, 95% CI: 0.11–0.76; frequently: OR: 0.15, 95% CI: 0.05–0.44) played a critical role. Depression was associated with after-work exercise, self-rated health, and occupational satisfaction for all PHWs. Additionally, living with family (OR: 0.51, 95% CI: 0.34–0.75) and organizational support satisfaction (satisfied: OR: 0.28, 95% CI: 0.19–0.42) were significant for urban practitioners.

**Conclusions:**

Risk factors such as occupational satisfaction, health, and family relations significantly influence PHW mental health in China, with notable differences by urbanicity. Tailored mental health interventions are recommended to address urban–rural disparities.

## Introduction

The mental health and well-being of medical staff have been largely neglected, despite mounting evidence of significant psychological burdens (McFarland *et al.*, 2019; Harvey *et al.*, 2021). Studies have consistently shown higher prevalence rates of mental health problems among physicians compared to age-matched controls (Baker *et al.*, 2017; McFarland *et al.*, 2019). For example, a landmark systematic review and meta-analysis in 2015 revealed a pooled prevalence of depression or depressive symptoms of 28.8% across 54 studies (Mata *et al.*, 2015). Subsequent research reported even higher rates of psychological issues, particularly during the Coronavirus Disease 2019 (COVID-19) pandemic (Khatun *et al.*, 2021; Adams *et al.*, 2023). Commentaries, such as those by Epstein and Privitera, have highlighted the alarmingly high rates of depression and anxiety disorders among physicians and the insufficient efforts to address these issues (Epstein and Privitera, 2019). Beyond documenting prevalence, it is imperative to identify the risk factors contributing to mental health challenges among medical staff to inform effective interventions.

Primary healthcare workers (PHWs), a critical yet underexamined group within the healthcare workforce, are especially vulnerable to being overlooked. By the end of 2023, China’s PHW workforce comprised 4.953 million individuals, representing 32.5% of the country’s medical personnel (National Health Commission of China, 2024). PHWs play an essential role in China’s healthcare system, delivering accessible and community-based healthcare, including both medical and public health services (Li *et al.*, 2020). During the COVID-19 pandemic, they were pivotal in vaccination campaigns, epidemiological investigations, and routine nucleic acid testing (Qian *et al.*, 2022). Despite their significance, nationwide studies on the mental health of PHWs in China remain scarce. While international research has documented anxiety and depression among PHWs in countries like Brazil and Turkey (de Souza Julio and Lourenção, 2021; Akova *et al.*, 2021), such studies are limited in China, particularly regarding comparisons between urban and rural areas.

Urban–rural disparities in healthcare resource distribution in China create distinct challenges for PHWs. Rural areas often face inadequate transportation, poor living conditions, and limited career development opportunities, contributing to unique stressors that may adversely affect PHWs’ mental health (Wang *et al.*, 2023). Conversely, urban PHWs often encounter intense work pressures, including workplace conflicts and incidents of verbal and physical abuse (Lafta and Falah, 2019; Zhang *et al.*, 2016). These contrasting conditions underscore the importance of examining the mental health risk factors of PHWs across urban and rural settings to develop targeted interventions.

This study aims to identify key factors influencing mental health challenges among PHWs in China. The conceptual framework is grounded in the Job Demand–Control–Support model and the Social Ecological Model, which emphasize that adequate workplace support plays a critical role in mental well-being, and that health outcomes are influenced by interacting factors across individual, interpersonal, community, and organizational levels (van der Doef and Maes, 1999; Golden and Earp, 2012). Guided by these frameworks and current research, we proposed five dimensions that have been reported to be associated with depression and anxiety among healthcare workers: individual factors (e.g., sex, age), family relations (e.g., living arrangement, marital status), work-related factors (e.g., workload, occupational category), occupational satisfaction (e.g., organizational support), and health factors (e.g., self-rated health, health behaviors) (Chan and Huak, 2004; Wong *et al.*, 2005; Bovier *et al.*, 2009; Brower and Riba, 2017; Moutier, 2018; Alsubaie *et al.*, 2019; Elbay *et al.*, 2020). By addressing these factors, this study seeks to provide policymakers with critical data to establish mental health assessments for PHWs, raise societal awareness, and offer insights for global innovations in mental health policies tailored to primary care.

## Methods

### Study design and participants

From May 1 to 31 October 2022, a nationwide survey was conducted across all 31 provinces of mainland China using a stratified four-stage random sampling method. Taiwan, Macao, and Hong Kong were excluded due to their distinct healthcare systems. The study first selected all 31 provinces and municipalities as sampling units. Within each province, one capital city and one administrative city were chosen. We chose a provincial capital city and an administrative city because the former can often represent a higher level of economic development in the province, while the randomly selected latter is often more general. During the actual implementation process, some cities refused to participate in the survey. To cope with this situation, we formed a new city sampling frame according to the principle of ‘difference in per capita GDP, population, and urbanization rate <5%’ and conducted re-sampling to replace the originally selected cities. Thirty-one capital cities and 31 administrative cities were selected. For urban samples, a community health center (CHC) and its affiliated stations were randomly selected in each capital city. For rural samples, one township hospital and its associated village clinics were selected in each administrative city. In the four municipalities of Beijing, Shanghai, Tianjin, and Chongqing, an additional CHC was chosen from both urban and suburban areas. Practical feasibility was considered alongside randomness to ensure accessibility to the selected CHCs or hospitals.

Due to urbanization, some township hospitals were reclassified as CHCs, and these facilities were included in the study. Overall, the survey covered 44 CHCs and 18 township health centers from 27 provinces and 4 municipalities. Eligible participants, including family physicians, nurses, public health professionals, pharmacists, and other medical staff working at these facilities, were all invited to participate in this survey. A total of 4,021 PHWs were included and 3,769 valid responses were used in the analysis (Figure S1).

### Measures and covariates

Mental health outcomes were assessed using validated measures. Depression symptoms were evaluated using the nine-item Patient Health Questionnaire (PHQ-9), with responses scored on a four-point Likert scale ranging from 0 (‘not at all’) to 3 (‘nearly every day’). Total scores ranged from 0 to 27, with higher scores indicating more severe symptoms. A cut-off score of 10 demonstrated high sensitivity (88%) and specificity (88%) for detecting major depression in medical populations. Anxiety symptoms were measured using the Generalized Anxiety Disorder (GAD-7) scale, which employs the same four-point Likert scale. A cut-off score of 10, based on Kroenke et al., yielded sensitivity and specificity of 89% and 82%, respectively, for GAD.

Participants provided detailed socio-demographic information, including sex, age, education level, household registration, monthly income, marital status, and living arrangements. Data on health and well-being included after-work exercise frequency, the presence of chronic conditions or physical disabilities, and self-rated health status. Work-related variables included professional rank, occupational category, practice location, years of employment, and self-rated work intensity. Occupational satisfaction was assessed on a three-point Likert scale, capturing perceptions of team support, organizational support, and overall satisfaction.

## Statistical analysis

Descriptive statistics were calculated to summarize participant characteristics, stratified by urban and rural groups, using R software (version 4.2.3). Categorical variables were expressed as frequencies and percentages, and chi-square tests were used to assess differences between groups. A two-sided *P*-value of less than 0.05 was considered statistically significant. To evaluate potential multicollinearity among risk factors, tolerance values and Variance Inflation Factors (VIF) were computed using the car package in R.

The Bayesian Additive Regression Tree (BART) model, implemented in the bartMachine package, was employed to identify key factors of anxiety and depression (Bleich and Kapelner, 2014). This machine learning approach is well-suited for detecting complex, non-linear relationships among variables and includes an integrated imputation mechanism for missing data (Chipman *et al.*, 2010; Hill, 2011). In addition, several studies have provided evidence that BART exhibits better predictive performance compared with many competing machine learning approaches, including Lasso, random forests, boosting models, and neural networks (Chipman *et al.*, 2010; Hill, 2011; Hu *et al.*, 2020). The model’s Variable Inclusion Proportion (VIP) scores were used to quantify the importance of covariates, with significance determined by simulating random distributions across 100 permutations (Hu *et al.*, 2020). Generally, the higher the VIP score (closer to 1), the more important the variable feature is in the model, and the more the model relies on this variable to make accurate predictions. Covariates with VIP scores exceeding the 95th percentile of the simulated distribution were considered statistically significant. The vertical line indicates the threshold level; variables exceeding this threshold are shown as solid dots, while unselected variables are shown as open dots.

To enhance the interpretability of findings, mixed-effects logistic regression analyses were conducted to quantify the effects of factors identified by the BART model on depression and anxiety, clustering at the provincial level. These analyses were performed separately for urban and rural subpopulations, providing tailored insights into the risk factors of mental health in each setting. Two sensitivity analyses included additional adjustment for demographic covariates and increased the variable inclusion threshold in the BART permutation procedure to the 99th percentile. As all questionnaire items were mandatory, no missing data were present.

## Results

### *Demographic characteristics of PHWs in* *China*

The demographic characteristics of the 3,769 PHWs included in the study are presented in Table 1. The mean age of participants was 37.38 years (standard deviation (SD) = 9.16), with 26.7% (1,006) working in urban areas and 73.3% (2,763) in rural areas. Overall, 21.2% (798) of participants were male, and the majority (59.2%, 2,230) held a bachelor’s degree or higher. Nearly half (41.6%, 1,569) worked in the eastern region of China. Most participants (93.8%, 3,535) were local residents born in the same region as their workplace, while 6.2% (234) were migrants. Monthly income ranged widely, with the largest proportion (43.0%, 1,620) reporting earnings between 3,000 and 5,000 RMB.

**Table 1.** Characteristics of participants (*N* = 3,769)

Variables		Total		Urban		Rural		*P*
		*N*	%	*N*	%	*N*	%	
Age (years)	≤30	974	25.8	289	28.7	685	24.8	0.002*
	31−40	1521	40.4	356	35.4	1165	42.2	
	41−50	880	23.4	246	24.5	634	22.9	
	>50	394	10.5	115	11.4	279	10.1	
Sex	Male	798	21.2	307	30.5	491	17.8	0.000**
	Female	2971	78.8	699	69.5	2272	82.2	
Educational attainment	Junior /high /technical school	407	10.8	229	22.8	178	6.4	0.000**
	Vocational school	1132	30.0	295	29.3	837	30.3	
	Bachelor’s degree or above	2230	59.2	482	47.9	1748	63.3	
Region	Eastern	1569	41.6	265	26.3	1304	47.2	0.000**
	Central	669	17.8	200	19.9	469	17	
	Western	1298	34.4	515	51.2	783	28.3	
	Northeastern	233	6.2	26	2.6	207	7.5	
Local residence	Local	3535	93.8	990	98.4	2545	92.1	0.000**
	Migrant	234	6.2	16	1.6	218	7.9	
Monthly income	<3,000 RMB	916	24.3	381	37.9	535	19.4	0.000**
	3,000–5,000 RMB	1620	43.0	415	41.3	1205	43.6	
	>5,000 RMB	1233	32.7	210	20.9	1023	37	
Marital status	Single	649	17.2	164	16.3	485	17.6	0.663
	Married	2926	77.6	789	78.4	2137	77.3	
	Divorced/widowed	194	5.2	53	5.3	141	5.1	
Family relationship	Dissatisfied	132	3.5	33	3.3	99	3.6	0.903
	Fair	577	15.3	155	15.4	422	15.3	
	Satisfied	3060	81.2	818	81.3	2242	81.1	
Living arrangement	Living alone	310	8.2	98	9.7	212	7.7	0.039*
	Living with family members	3281	87.1	870	86.5	2411	87.3	
	Living with non-family members	178	4.7	38	3.8	140	5.1	
After-work exercise	Never	161	4.3	27	2.7	134	4.8	0.000**
	Rarely	533	14.1	110	10.9	423	15.3	
	Occasionally	1684	44.7	462	45.9	1222	44.2	
	Sometimes	905	24.0	268	26.6	637	23.1	
	Frequently	486	12.9	139	13.8	347	12.6	
Number of current chronic illnesses	0	2936	77.9	812	80.7	2124	76.9	0.038*
	1	598	15.9	142	14.1	456	16.5	
	2 or more	235	6.2	52	5.2	183	6.6	
Physical disabilities	No	3680	97.6	979	97.3	2701	97.8	0.431
	Yes	89	2.4	27	2.7	62	2.2	
Self-rated health status	Poor	397	10.5	72	7.2	325	11.8	0.000**
	Fair	2082	55.2	498	49.5	1584	57.3	
	Good	1290	34.2	436	43.3	854	30.9	
Professional rank	Unranked	548	14.5	246	24.5	302	10.9	0.000**
	Junior	1686	44.7	436	43.3	1250	45.2	
	Intermediate	1225	32.5	251	25	974	35.3	
	Senior	310	8.2	73	7.3	237	8.6	
Occupation category	Physician	1174	31.2	429	42.6	745	27	0.000**
	Nurse	1282	34.0	253	25.1	1029	37.2	
	Public health professional	253	6.7	62	6.2	191	6.9	
	Administrative staff	221	5.9	47	4.7	174	6.3	
	Others	839	22.3	215	21.4	624	22.6	
Years of employment in current organization	5 years or less	1468	39.0	323	32.1	1145	41.4	0.000**
	6–10 years	872	23.1	224	22.3	648	23.5	
	11–20 years	859	22.8	254	25.2	605	21.9	
	Over 20 years	570	15.1	205	20.4	365	13.2	
Self-rated work intensity	Low	19	0.5	8	0.8	11	0.4	0.000**
	Moderate	1145	30.4	366	36.4	779	28.2	
	High	2605	69.1	632	62.8	1973	71.4	
Team support	Dissatisfied	290	7.7	88	8.7	202	7.3	0.201
	Moderate	1326	35.2	363	36.1	963	34.9	
	Satisfied	2153	57.1	555	55.2	1598	57.8	
Organizational support	Dissatisfied	352	9.3	93	9.2	259	9.4	0.243
	Moderate	1417	37.6	400	39.8	1017	36.8	
	Satisfied	2000	53.1	513	51	1487	53.8	
Self-rated job satisfaction	Dissatisfied	329	8.7	91	9	238	8.6	0.798
	Moderate	1636	43.4	442	43.9	1194	43.2	
	Satisfied	1804	47.9	473	47	1331	48.2	
Anxiety	<10 Minimal-mild anxiety	3165	84.0	2314	83.8	851	84.6	0.533
	≥10 Moderate-severe anxiety	604	16.0	449	16.2	155	15.4	
Depression	<10 Minimal-mild depression	3079	81.7	2250	81.4	829	82.4	0.495
	≥10 Moderate-severe depression	690	18.3	513	18.6	177	17.6	

* *P* < 0.05.

** *P* < 0.01.

Significant differences were observed between urban and rural groups in age, sex, educational attainment, geographic region, residency status, monthly income, living arrangement, after-work exercise frequency, number of chronic illnesses, self-rated health status, professional rank, occupational category, years of employment, and self-rated work intensity (all *P* < 0.05).

### Key factors identified by BART analysis

**Figure 1.**
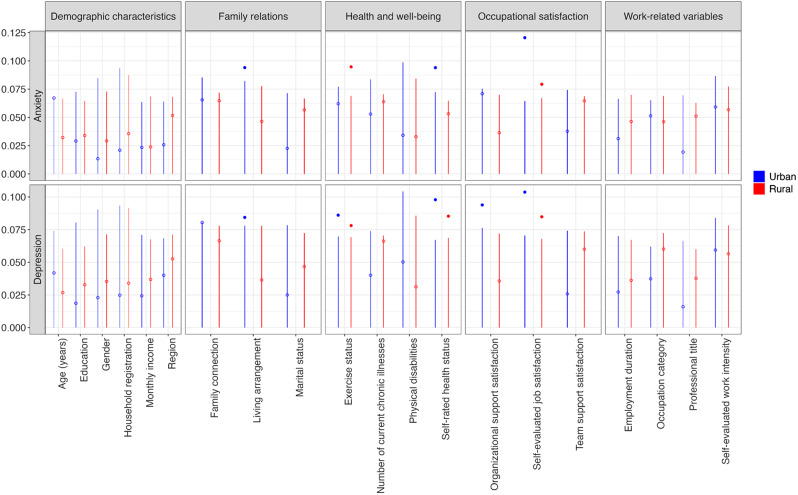
Distributions of Variable Inclusion Proportions (VIP).

Figure 1 illustrates the variable importance scores derived from the BART model. For anxiety, four key factors were identified: living arrangement (cutoff = 0.082, VIP = 0.094), self-rated health status (cutoff = 0.072, VIP = 0.094), job satisfaction in urban areas (cutoff = 0.064, VIP = 0.120), and after-work exercise in rural areas (cutoff = 0.069, VIP = 0.095).

For depression, eight factors were identified, including living arrangement (cutoff = 0.078, VIP = 0.084), organizational support satisfaction in urban areas (cutoff = 0.076, VIP = 0.094), after-work exercise (cutoffs = 0.070 and 0.069; VIPs = 0.086 and 0.078), self-rated health status (cutoffs = 0.067 and 0.069; VIPs = 0.098 and 0.085), and job satisfaction (cutoffs = 0.071 and 0.068; VIPs = 0.104 and 0.085) across urban and rural settings (Tables S1–S4). When we increased the variable inclusion threshold from 95th to 99th percentile, self-evaluated job satisfaction remained a significant factor in both urban and rural settings (Tables S5–S9).

### Risk factors for anxiety by urbanicity

No multicollinearity concerns were identified (Tolerance >0.1, VIF <10; Supplementary Tables S9–S12). Among urban PHWs, moderate jobS satisfaction (OR = 0.24, 95% CI, 0.17–0.33) and high job satisfaction (OR = 0.10, 95% CI, 0.07–0.15) were associated with lower anxiety. Living with family (OR = 0.42, 95% CI, 0.28–0.62) and self-rated health as fair (OR = 0.31, 95% CI, 0.23–0.42) or good (OR = 0.13, 95% CI, 0.09–0.20) were also protective against anxiety (all *P* < 0.005).

**Figure 2.**
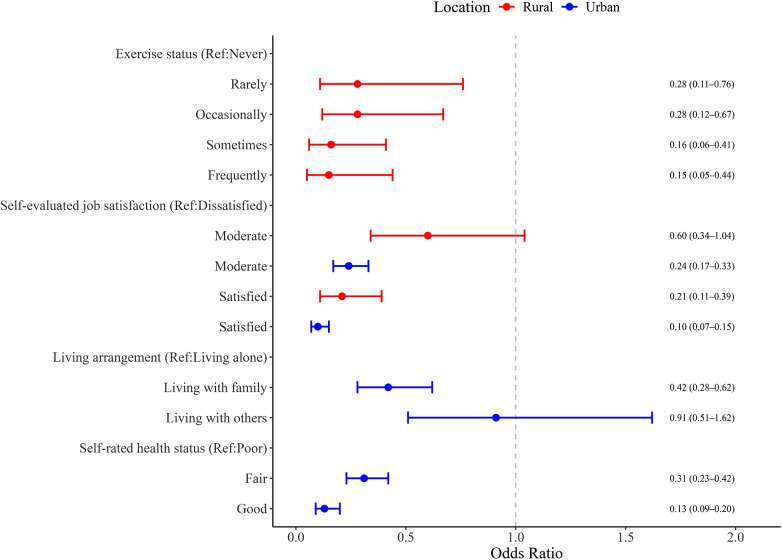
Significant factors of anxiety among primary healthcare workers in rural and urban areas.

In rural PHWs, engaging in after-work exercise was strongly associated with reduced anxiety. Compared to those who never exercised, individuals who rarely (OR = 0.28, 95% CI, 0.11–0.76), occasionally (OR = 0.28, 95% CI, 0.12–0.67), sometimes (OR = 0.16, 95% CI, 0.06–0.41), or frequently (OR = 0.15, 95% CI, 0.05–0.44) exercised had significantly lower odds of anxiety. High job satisfaction (OR = 0.21, 95% CI, 0.11–0.39) was also protective. These results are visualized in Fig. 2.

### Risk factors for depression by urbanicity

**Figure 3.**
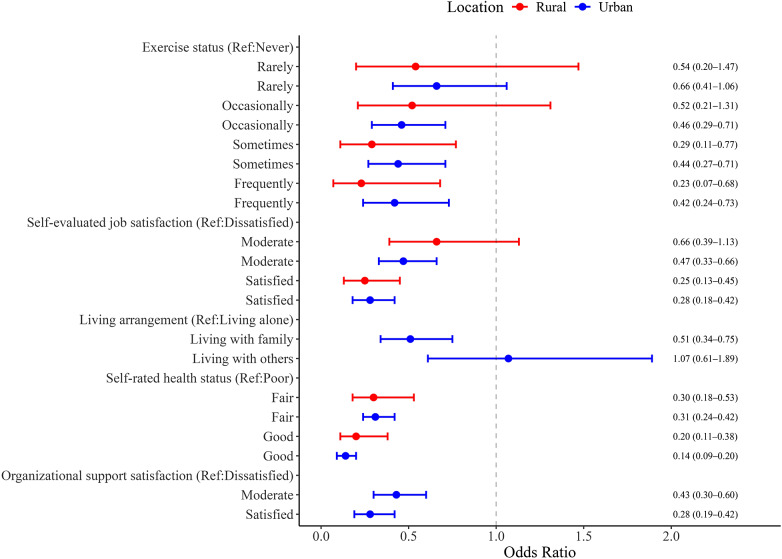
Significant factors of depression among primary healthcare workers in rural and urban areas.

Figure 3 highlights the risk factors of depression. Among urban PHWs, engaging in after-work exercise occasionally (OR = 0.46, 95% CI, 0.29–0.71), sometimes (OR = 0.44, 95% CI, 0.27–0.71), or frequently (OR = 0.42, 95% CI, 0.24–0.73) was associated with lower depression risk. Moderate (OR = 0.43, 95% CI, 0.30–0.60) and high job satisfaction (OR = 0.28, 95% CI, 0.19–0.42), living with family (OR = 0.51, 95% CI, 0.34–0.75), and self-rated health as fair (OR = 0.31, 95% CI, 0.24–0.42) or good (OR = 0.14, 95% CI, 0.09–0.20) were similarly protective. Satisfaction with organizational support (moderate: OR = 0.43, 95% CI, 0.30–0.60; high: OR = 0.28, 95% CI, 0.19–0.42) also significantly reduced depression risk.

Among rural PHWs, after-work exercise remained a critical factor, with lower depression risk observed for those exercising sometimes (OR = 0.29, 95% CI, 0.11–0.77) or frequently (OR = 0.23, 95% CI, 0.07–0.68). High job satisfaction (OR = 0.25, 95% CI, 0.13–0.45) and self-rated health as fair (OR = 0.30, 95% CI, 0.18–0.53) or good (OR = 0.20, 95% CI, 0.11–0.38) were also protective against depression. Results remained consistent after adjusting for demographic variables (Supplementary Tables S13–S16).

## Discussion

This national survey was conducted at a pivotal time, immediately following the COVID-19 pandemic, to examine factors influencing the mental health of PHWs in China. Using a permutation-based variable selection technique, the key factors of anxiety and depression were identified. Notably, demographic and workload-related variables, commonly emphasized in previous studies, were not significant factors in this study (Győrffy *et al.*, 2016; Moutier, 2018). These findings align with, yet differ in certain respects from, existing literature on the psychological health of medical personnel, potentially reflecting the unique context of the pandemic and the characteristics of PHWs surveyed (Selamu *et al.*, 2019).

Self-rated job satisfaction emerged as a critical factor of anxiety and depression across both urban and rural settings, consistent with extensive evidence highlighting its positive impact on mental well-being among physicians globally, including studies in the United States, the United Kingdom, Japan, and Nigeria (Ramirez *et al.*, 1996; Williams *et al.*, 2000, 2002; Tokuda *et al.*, 2009; Bello *et al.*, 2013; Kamimura *et al.*, 2018). Although research specifically focused on PHWs is limited, findings consistently underscore the positive impact of job satisfaction (Unrath *et al.*, 2012; Yilmaz, 2018; Al-Wotayan *et al.*, 2019). Interestingly, organizational support satisfaction was uniquely associated with depression among urban PHWs. This finding may reflect the heightened pressures faced by urban PHWs during the pandemic, including serving larger populations, managing higher infection rates, and facilitating extensive vaccination campaigns (Zulu *et al.*, 2025). The increased reliance of urban PHWs on institutional support, such as access to personal protective equipment, underscores the importance of organizational backing in mitigating mental health challenges in urban settings (Ni *et al.*, 2021).

Contrary to prior research, workload-related variables were not significant risk factors of mental health in this study. This discrepancy may be attributable to the timing of the survey, conducted during the final phase of the pandemic when workload pressures had eased compared to the pandemic’s earlier years (Knight *et al.*, 2023). Self-rated health and after-work exercise were also identified as significant predictors of anxiety and depression. Self-rated health, a subjective measure linked to biological indicators such as mortality, has been consistently associated with mental health outcomes (Fayers and Sprangers, 2002; Jylhä, 2009). For instance, studies have demonstrated that poor self-rated health predicts depressive symptoms, even after adjusting for covariates (Ambresin *et al.*, 2014). Similarly, physical activity and exercise have been widely recognized as protective factors for mental health, though findings within medical populations have been mixed (Han *et al.*, 2018; Östberg and Nordin, 2022). For example, research in southern China found an association between poor self-rated health, lack of exercise, and higher rates of anxiety and depression (Rebar *et al.*, 2015; Gong *et al.*, 2014). Conversely, a study among physician assistant students in the United States reported no significant relationship between exercise and mental health outcomes (Wright, 2023). Our study potentially reflects the heightened need for physical well-being among medical staff managing infectious disease emergencies.

Future research should further explore the mechanisms underlying these relationships. Family relationships, particularly living arrangements, were significantly associated with anxiety and depression among urban PHWs. While family support has been well-documented as a protective factor for mental health in adolescents and the elderly (Lau and Kwok, 2000; Bögels and Brechman-Toussaint, 2006; Guerrero-Muñoz *et al.*, 2020), its role in medical staff is less studied. Our findings align with previous research indicating that family environment and social support were negatively correlated with symptoms of anxiety and depression among healthcare workers in urban areas during the COVID-19 pandemic (Fang *et al.*, 2021; Nie *et al.*, 2023). However, this association was not observed among rural PHWs, likely reflecting the broader and more diverse social networks in rural China, including community and clan-based support systems, which may reduce reliance on nuclear family support (Xu *et al.*, 2023).

Our findings indicate both shared and distinct factors for anxiety and depression among PHWs. For shared factors, better self-rated health and more frequent after-work exercise were consistently associated with lower odds of mental illness. This is consistent with the current research findings that self-rated health and healthy behaviors are positively associated with mental health, although these studies have a broader population and are not limited to primary care workers (Kouvonen *et al.*, 2021; Peleg and Nudelman, 2021; Ross *et al.*, 2023). However, differences were also observed: living with family was particularly protective against anxiety, especially among urban healthcare workers, and depression was more closely related to occupational satisfaction and perceived organizational support. These similar and different factors suggest that future intervention strategies for primary care workers’ mental health should also be different. For example, workplace support programs may be more effective in alleviating depression, while family support programs may be more effective in alleviating anxiety.

It should be noted that the data in this study were collected during the COVID-19 pandemic, which may have amplified the levels of anxiety and depression observed. However, several structural factors identified in this study – such as self-rated health status, exercise after work, job satisfaction, organizational support satisfaction, and living arrangement – are not specific to the pandemic but are factors that continue to impact mental health (Liu *et al.*, 2023; Turgut *et al.*, 2024; Zheng and Zhang, 2024; Li *et al.*, 2025). Therefore, the findings are not only relevant for guiding mental health support during the pandemic but also provide important evidence for developing long-term mental health promotion strategies in the post-pandemic era.

Although this study provides valuable insights, several limitations should be acknowledged. First, in our study, we employed a stratified four-stage sampling method. However, in practice, when encountering cities or institutions that refused to participate, we regenerated the sampling frame, which differed slightly from the original research design. Second, the cross-sectional design precludes causal inferences and limits the ability to capture changes in mental health risk factors over time. Longitudinal studies are needed to explore how these risk factors evolve. Third, while this study identified significant factors, it did not investigate the intermediate mechanisms through which these factors influence mental health. Future research should examine these pathways and consider intervention strategies to mitigate mental health risks among PHWs.

## Conclusion

This study represents one of the few nationwide surveys in China focusing on the mental health of PHWs who serve on the front lines of community healthcare. Conducted immediately following the COVID-19 pandemic, the survey encompassed urban and rural areas across all 31 provinces of mainland China, providing a comprehensive analysis of mental health risk factors using a machine learning approach. Key factors identified included job satisfaction, self-rated health, and family relationships, while demographic and workload-related variables were not significant factors. Risk factors of anxiety and depression varied by urbanicity. Occupational satisfaction and self-rated health were significant in both urban and rural settings, while organizational support satisfaction and living arrangements were uniquely relevant to urban practitioners. These findings highlight the importance of tailored mental health interventions that address the distinct needs of urban and rural PHWs. Given the significant urban–rural disparities in China, targeted strategies are essential to ensure equitable mental health support for PHWs and to sustain the critical role they play in community healthcare delivery.

## Supporting information

10.1017/S2045796025100425.sm001 Huang et al. supplementary material 1 Huang et al. supplementary material

10.1017/S2045796025100425.sm002 Huang et al. supplementary material 2 Huang et al. supplementary material

## Data Availability

Datasets generated and analysed during the current study are not publicly available, as this was not included in the consent process, but anonymized datasets can be made available from the corresponding author on reasonable request by email.

## Supplementary material

The supplementary material for this article can be found at https://doi.org/10.1017/S2045796025100425.
