# Supplementary material for: Urban–rural differences in determinants of mental health among primary healthcare workers in China
Source: Epidemiol Psychiatr Sci. 2026 Jan 7;35:e4. doi: 10.1017/S2045796025100425 (PMC12816933; doi:10.1017/S2045796025100425)
Supplement: Huang et al. supplementary material 1 — Huang et al. supplementary material [file S2045796025100425sup001.docx]

**Supplementary material：**

**
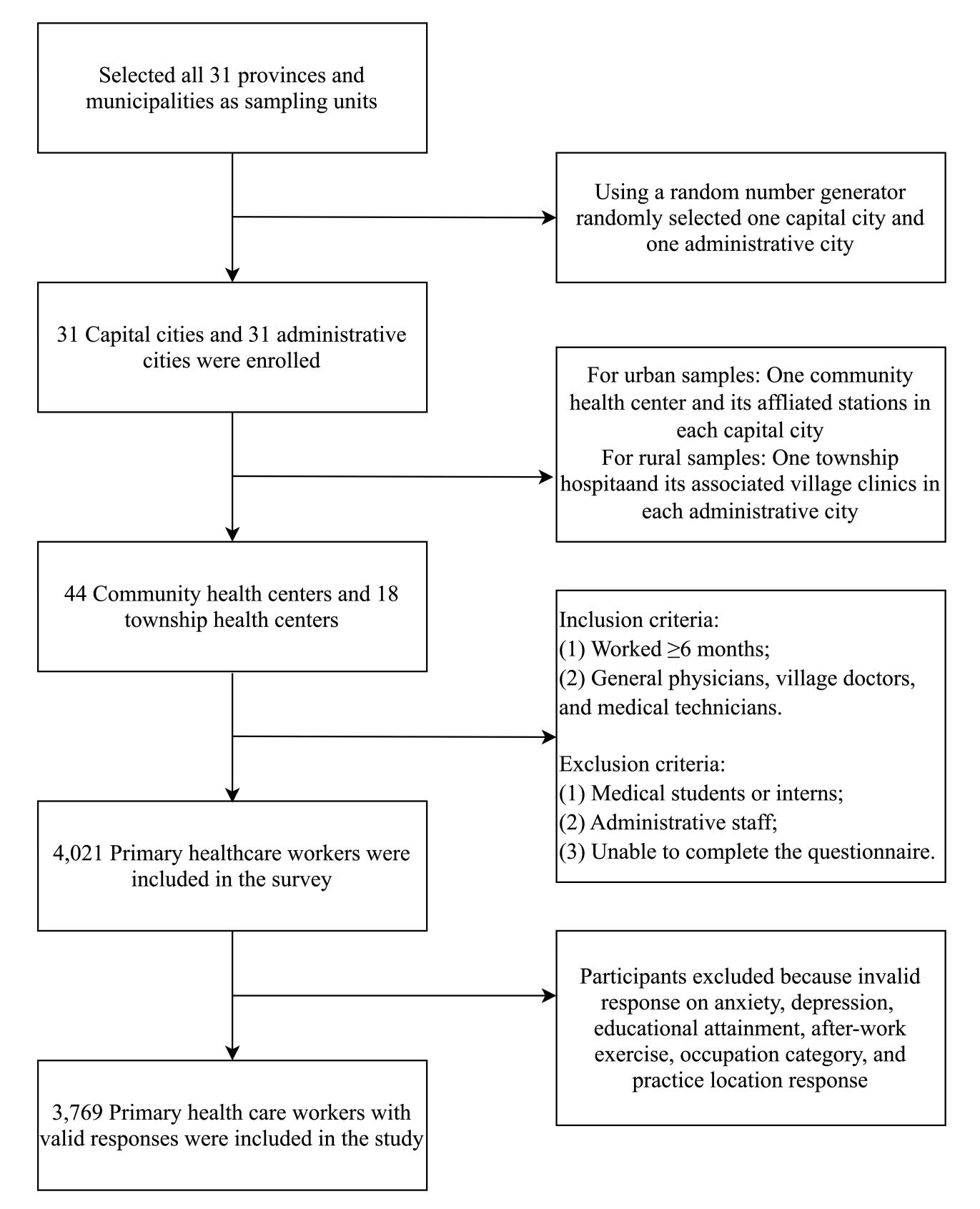
**

Figure S1. Flow chart of participant inclusion and exclusion among primary healthcare workers in China

The survey included 44 community health centers and 18 township health centers across 27 provinces and 4 municipalities in China. A total of 4,021 primary healthcare workers responded. After applying the inclusion and exclusion criteria, 3,769 valid responses were included in the analysis.

**Figure Captions:**

Figure 1: Predictors were grouped into five conceptual domains: demographic characteristics, family relations, health and well-being, occupational satisfaction, and work-related variables. Within each domain, potential determinants are displayed according to their Variable Inclusion Proportion (VIP) scores derived from the Bayesian Additive Regression Tree (BART) model. Blue lines indicate results for urban participants, while red lines indicate results for rural participants.

Figure 2: Logistic regression analyses based on BART-identified predictors were conducted separately for urban (blue) and rural (red) primary healthcare workers to examine their associations with anxiety. Odds ratios (ORs) with 95% confidence intervals (CIs) are presented.

Figure 3: Logistic regression analyses based on BART-identified predictors were conducted separately for urban (blue) and rural (red) primary healthcare workers to examine their associations with depression. Odds ratios (ORs) with 95% confidence intervals (CIs) are presented.

**Table S1 Urban area primary healthcare workers’ anxiety cutoff value and variable inclusion proportion.**

|  | **Cutoff** | **Proportions** |
| --- | --- | --- |
| Age (years) | 0.06738254 | 0.067192297 |
| Number of current chronic illnesses | 0.083626744 | 0.052891023 |
| Years of employment in current organization | 0.066500664 | 0.031176342 |
| Educational attainment | 0.072624731 | 0.029110929 |
| After-work exercise | 0.077120622 | 0.062119259 |
| Family relationship | 0.085301444 | 0.065518717 |
| Sex | 0.084542942 | 0.013413855 |
| Local residence | 0.093542896 | 0.020948945 |
| Living arrangement | 0.082112901 | 0.094023882 |
| Marital status | 0.071532748 | 0.022611732 |
| Monthly income | 0.063557183 | 0.023451629 |
| Occupation category | 0.065124185 | 0.051356595 |
| Organizational support | 0.07533624 | 0.070918273 |
| Physical disabilities | 0.098604167 | 0.034193665 |
| Professional rank | 0.069490443 | 0.019328458 |
| Region | 0.063988289 | 0.025723353 |
| Self-rated job satisfaction | 0.064370605 | 0.120498139 |
| Self-rated work intensity | 0.086521146 | 0.059187699 |
| Self-rated health status | 0.072347454 | 0.093998145 |
| Team support | 0.07424597 | 0.037728853 |

**Table S2 Rural area primary healthcare workers’ anxiety cutoff value and variable inclusion proportion.**

|  | **Cutoff** | **Proportions** |
| --- | --- | --- |
| Age (years) | 0.066737963 | 0.032196874 |
| Number of current chronic illnesses | 0.070420492 | 0.063901645 |
| Years of employment in current organization | 0.069999389 | 0.046351995 |
| Educational attainment | 0.064560176 | 0.033969166 |
| After-work exercise | 0.06903111 | 0.094719011 |
| Family relationship | 0.071748115 | 0.064701888 |
| Sex | 0.072970764 | 0.029255119 |
| Local residence | 0.087464164 | 0.035678879 |
| Living arrangement | 0.077536675 | 0.046478146 |
| Marital status | 0.066784336 | 0.056604917 |
| Monthly income | 0.068539849 | 0.023876918 |
| Occupation category | 0.06887838 | 0.046201925 |
| Organizational support | 0.070091236 | 0.036447698 |
| Physical disabilities | 0.084290797 | 0.032861947 |
| Professional rank | 0.062666862 | 0.051064815 |
| Region | 0.068315682 | 0.051691771 |
| Self-rated job satisfaction | 0.067066241 | 0.079329456 |
| Self-rated work intensity | 0.077420282 | 0.056831308 |
| Self-rated health status | 0.06466601 | 0.053207441 |
| Team support | 0.068812022 | 0.06462908 |

**Table S3 Urban area primary healthcare workers’ depression cutoff value and variable inclusion proportion.**

|  | **Cutoff** | **Proportions** |
| --- | --- | --- |
| Age (years) | 0.073990659 | 0.041838413 |
| Number of current chronic illnesses | 0.073884421 | 0.040076011 |
| Years of employment in current organization | 0.070160999 | 0.027241594 |
| Educational attainment | 0.08054681 | 0.018642277 |
| After-work exercise | 0.069735473 | 0.08607177 |
| Family relationship | 0.080616096 | 0.080483781 |
| Sex | 0.090330767 | 0.022887977 |
| Local residence | 0.093225138 | 0.024836046 |
| Living arrangement | 0.077933772 | 0.084338854 |
| Marital status | 0.078407354 | 0.025002422 |
| Monthly income | 0.070911215 | 0.024298163 |
| Occupation category | 0.062111972 | 0.037344161 |
| Organizational support | 0.076304899 | 0.093901496 |
| Physical disabilities | 0.104250924 | 0.050260473 |
| Professional rank | 0.066443021 | 0.015982417 |
| Region | 0.068349966 | 0.040000341 |
| Self-rated job satisfaction | 0.07050096 | 0.103713216 |
| Self-rated work intensity | 0.083972795 | 0.05936737 |
| Self-rated health status | 0.06711584 | 0.097887467 |
| Team support | 0.0741286 | 0.025825751 |

**Table S4 Rural area primary healthcare workers’ depression cutoff value and variable inclusion proportion.**

|  | **Cutoff** | **Proportions** |
| --- | --- | --- |
| Age (years) | 0.060272249 | 0.026818083 |
| Number of current chronic illnesses | 0.070494825 | 0.066170488 |
| Years of employment in current organization | 0.067177315 | 0.036222354 |
| Educational attainment | 0.062042746 | 0.03279788 |
| After-work exercise | 0.069216684 | 0.07811829 |
| Family relationship | 0.078087372 | 0.066451101 |
| Sex | 0.071314687 | 0.03535299 |
| Local residence | 0.091592828 | 0.033986695 |
| Living arrangement | 0.077880523 | 0.03645089 |
| Marital status | 0.072359346 | 0.046763364 |
| Monthly income | 0.06771841 | 0.036849278 |
| Occupation category | 0.07235105 | 0.060130841 |
| Organizational support | 0.07198687 | 0.03571701 |
| Physical disabilities | 0.085615762 | 0.031232608 |
| Professional rank | 0.060001688 | 0.037694501 |
| Region | 0.071257236 | 0.052604839 |
| Self-rated job satisfaction | 0.067926728 | 0.084809381 |
| Self-rated work intensity | 0.078244505 | 0.056519057 |
| Self-rated health status | 0.06862901 | 0.085305357 |
| Team support | 0.073702679 | 0.060004993 |

**Table S5. Variable inclusion proportions for anxiety in urban primary healthcare workers based on the 99th percentile cutoff threshold.**

|  | **Cutoff** | **Proportions** |
| --- | --- | --- |
| Age (years) | 0.0758843308210076 | 0.0663751158752249 |
| Number of current chronic illnesses | 0.0909196647454126 | 0.0536123202515173 |
| Years of employment in current organization | 0.079159139651761 | 0.0308730252894334 |
| Educational attainment | 0.0812130290799161 | 0.0307519946272435 |
| After-work exercise | 0.0813011135840254 | 0.0652313362122699 |
| Family relationship | 0.0905627520917991 | 0.0624594354664374 |
| Sex | 0.0964533357772543 | 0.0166346221288469 |
| Local residence | 0.098083110873735 | 0.0207984917407542 |
| Living arrangement | 0.102977729176983 | 0.0953246128045213 |
| Marital status | 0.0913859935840513 | 0.0232419355676064 |
| Monthly income | 0.080041028760322 | 0.0208558742430974 |
| Occupation category | 0.074107763155034 | 0.049399457478889 |
| Organizational support | 0.076032027102247 | 0.0749869874799772 |
| Physical disabilities | 0.102718057537771 | 0.0372196071700494 |
| Professional rank | 0.0839718670638518 | 0.0179618560637733 |
| Region | 0.0855455433738633 | 0.0233832619689176 |
| Self-rated job satisfaction | 0.0851295934738441 | 0.120928318626321 |
| Self-rated work intensity | 0.0889708003058764 | 0.0599899187141837 |
| Self-rated health status | 0.0973571371297337 | 0.0897563564051618 |
| Team support | 0.0835991904575568 | 0.0402154718857747 |

**Table S6. Variable inclusion proportions for anxiety in rural primary healthcare workers based on the 99th percentile cutoff threshold.**

|  | **Cutoff** | **Proportions** |
| --- | --- | --- |
| Age (years) | 0.0797036922181235 | 0.0684051965224415 |
| Number of current chronic illnesses | 0.100408225737869 | 0.0505520549281262 |
| Years of employment in current organization | 0.0981044761331233 | 0.0324553489567186 |
| Educational attainment | 0.0831447969863057 | 0.0307484593000614 |
| After-work exercise | 0.0809125845608342 | 0.0619249462850787 |
| Family relationship | 0.0926322486125713 | 0.065603445911543 |
| Sex | 0.107421526108177 | 0.0187632058463671 |
| Local residence | 0.0972664021997642 | 0.0130081112234012 |
| Living arrangement | 0.0967177682456055 | 0.0929374498866946 |
| Marital status | 0.0875967506293072 | 0.0238283153221269 |
| Monthly income | 0.0816606438876181 | 0.0220894059184921 |
| Occupation category | 0.0855462466313045 | 0.0521309670455537 |
| Organizational support | 0.0816754778032494 | 0.0756071856191697 |
| Physical disabilities | 0.10794894397187 | 0.0372878153287958 |
| Professional rank | 0.0745541249638153 | 0.0167267923028496 |
| Region | 0.0809066501601912 | 0.0242386002517139 |
| Self-rated job satisfaction | 0.0774025032147501 | 0.126002006062603 |
| Self-rated work intensity | 0.0942472560971562 | 0.0576123575167656 |
| Self-rated health status | 0.0950082451131075 | 0.0898144675111207 |
| Team support | 0.079814818997749 | 0.0402638682603771 |

**Table S7. Variable inclusion proportions for depression in urban primary healthcare workers based on the 99th percentile cutoff threshold.**

|  | **Cutoff** | **Proportions** |
| --- | --- | --- |
| Age (years) | 0.0751104410873178 | 0.0402200672350326 |
| Number of current chronic illnesses | 0.0948781046072461 | 0.0365681012893434 |
| Years of employment in current organization | 0.0715515021942039 | 0.0285388063908987 |
| Educational attainment | 0.0922418712546215 | 0.0213810493516412 |
| After-work exercise | 0.0835334758012072 | 0.0846849929718871 |
| Family relationship | 0.0918662849564209 | 0.0802943924696099 |
| Sex | 0.109100744281174 | 0.0204951522053971 |
| Local residence | 0.104264088084239 | 0.0278361290670042 |
| Living arrangement | 0.108502280981302 | 0.0887045624218482 |
| Marital status | 0.0900164077952984 | 0.0225606045132663 |
| Monthly income | 0.0799034544313977 | 0.0234230556636781 |
| Occupation category | 0.0836747631681869 | 0.0397769587621132 |
| Organizational support | 0.072058006662382 | 0.093243701939079 |
| Physical disabilities | 0.109716812034423 | 0.0526163735815173 |
| Professional rank | 0.0834040675901703 | 0.0153652355770331 |
| Region | 0.0654754795651589 | 0.0385998492879127 |
| Self-rated job satisfaction | 0.0922608713564852 | 0.105222695676336 |
| Self-rated work intensity | 0.0999773186750388 | 0.0607725840231167 |
| Self-rated health status | 0.0936609106370382 | 0.0941137007984171 |
| Team support | 0.0848091130434002 | 0.0255819867748678 |

**Table S8. Variable inclusion proportions for depression in rural primary healthcare workers based on the 99th percentile cutoff threshold.**

|  | **Cutoff** | **Proportions** |
| --- | --- | --- |
| Age (years) | 0.0754541993209199 | 0.0242974911665305 |
| Number of current chronic illnesses | 0.084025461205256 | 0.0686884033569495 |
| Years of employment in current organization | 0.0762371821228373 | 0.0367339537237991 |
| Educational attainment | 0.0757425331327452 | 0.0287576391823504 |
| After-work exercise | 0.0984150060337452 | 0.0784292418596038 |
| Family relationship | 0.08402469438463 | 0.0679903789225123 |
| Sex | 0.0809553634975127 | 0.0359138855231393 |
| Local residence | 0.0894913648484567 | 0.0333155100997145 |
| Living arrangement | 0.0865643187870896 | 0.041516456811136 |
| Marital status | 0.086660341204581 | 0.0444869903127268 |
| Monthly income | 0.0704752811762325 | 0.0377628321492986 |
| Occupation category | 0.0755988997739908 | 0.064039576320295 |
| Organizational support | 0.0848891761507828 | 0.0348236726240867 |
| Physical disabilities | 0.0884382338797875 | 0.0300741156185002 |
| Professional rank | 0.0760728964258377 | 0.0369126204426767 |
| Region | 0.0892452335236674 | 0.0502056079570652 |
| Self-rated job satisfaction | 0.075304562779803 | 0.0838016038168795 |
| Self-rated work intensity | 0.0938152948411927 | 0.0599515624457562 |
| Self-rated health status | 0.0762722705059576 | 0.0842789958563683 |
| Team support | 0.0852615150127547 | 0.0580194618106113 |

**Table S9 Multicollinearity test of anxiety risk factors for rural area primary healthcare workers.**

|  | **Tolerance** | **VIF** |
| --- | --- | --- |
| Age (years) | 0.341 | 2.933 |
| Sex | 0.826 | 1.211 |
| Educational attainment | 0.591 | 1.692 |
| Region | 0.911 | 1.098 |
| Local residence | 0.965 | 1.036 |
| Monthly income | 0.632 | 1.582 |
| Marital status | 0.75 | 1.333 |
| Family relationship | 0.915 | 1.093 |
| Living arrangement | 0.939 | 1.065 |
| After-work exercise | 0.841 | 1.189 |
| Number of current chronic illnesses | 0.806 | 1.240 |
| Physical disabilities | 0.963 | 1.039 |
| Self-rated health status | 0.732 | 1.365 |
| Professional rank | 0.610 | 1.639 |
| Occupation category | 0.961 | 1.041 |
| Practice Location | 0.438 | 2.281 |
| Years of employment in current organization | 0.873 | 1.146 |
| Self-rated work intensity | 0.305 | 3.275 |
| Team support | 0.307 | 3.263 |
| Organizational support | 0.617 | 1.621 |

**Table S10 Multicollinearity test of depression risk factors for rural area primary healthcare workers.**

|  | **Tolerance** | **VIF** |
| --- | --- | --- |
| Age (years) | 0.341 | 2.933 |
| Sex | 0.826 | 1.211 |
| Educational attainment | 0.591 | 1.692 |
| Region | 0.911 | 1.098 |
| Local residence | 0.965 | 1.036 |
| Monthly income | 0.632 | 1.582 |
| Marital status | 0.75 | 1.333 |
| Family relationship | 0.915 | 1.093 |
| Living arrangement | 0.939 | 1.065 |
| After-work exercise | 0.841 | 1.189 |
| Number of current chronic illnesses | 0.806 | 1.24 |
| Physical disabilities | 0.963 | 1.039 |
| Self-rated health status | 0.732 | 1.365 |
| Professional rank | 0.61 | 1.639 |
| Occupation category | 0.961 | 1.041 |
| Practice Location | 0.438 | 2.281 |
| Years of employment in current organization | 0.873 | 1.146 |
| Self-rated work intensity | 0.305 | 3.275 |
| Team support | 0.307 | 3.263 |
| Organizational support | 0.617 | 1.621 |

**Table S11 Multicollinearity test of anxiety risk factors for urban area primary healthcare workers.**

|  | **Tolerance** | **VIF** |
| --- | --- | --- |
| Age (years) | 0.378 | 2.644 |
| Sex | 0.937 | 1.067 |
| Educational attainment | 0.748 | 1.337 |
| Region | 0.881 | 1.136 |
| Local residence | 0.943 | 1.06 |
| Monthly income | 0.667 | 1.498 |
| Marital status | 0.769 | 1.3 |
| Family relationship | 0.934 | 1.07 |
| Living arrangement | 0.983 | 1.017 |
| After-work exercise | 0.861 | 1.161 |
| Number of current chronic illnesses | 0.781 | 1.281 |
| Physical disabilities | 0.966 | 1.035 |
| Self-rated health status | 0.738 | 1.354 |
| Professional rank | 0.541 | 1.85 |
| Occupation category | 0.918 | 1.09 |
| Practice Location | 0.535 | 1.869 |
| Years of employment in current organization | 0.905 | 1.105 |
| Self-rated work intensity | 0.374 | 2.673 |
| Team support | 0.337 | 2.969 |
| Organizational support | 0.564 | 1.773 |

**Table S12 Multicollinearity test of depression risk factors for urban area primary healthcare workers.**

|  | **Tolerance** | **VIF** |
| --- | --- | --- |
| Age (years) | 0.378 | 2.644 |
| Sex | 0.937 | 1.067 |
| Educational attainment | 0.748 | 1.337 |
| Region | 0.881 | 1.136 |
| Local residence | 0.943 | 1.060 |
| Monthly income | 0.667 | 1.498 |
| Marital status | 0.769 | 1.300 |
| Family relationship | 0.934 | 1.07 |
| Living arrangement | 0.983 | 1.017 |
| After-work exercise | 0.861 | 1.161 |
| Number of current chronic illnesses | 0.781 | 1.281 |
| Physical disabilities | 0.966 | 1.035 |
| Self-rated health status | 0.738 | 1.354 |
| Professional rank | 0.541 | 1.85 |
| Occupation category | 0.918 | 1.09 |
| Practice Location | 0.535 | 1.869 |
| Years of employment in current organization | 0.905 | 1.105 |
| Self-rated work intensity | 0.374 | 2.673 |
| Team support | 0.337 | 2.969 |
| Organizational support | 0.564 | 1.773 |

**Table S13 Significant factors of anxiety among primary healthcare workers in rural area, including demographic characteristics.**

| Characteristic | OR (95% CI) | *P*-value |
| --- | --- | --- |
| Exercise status (Ref: Never) |  |  |
| Rarely | 0.24 (0.09, 0.65) | 0.004 |
| Occasionally | 0.23 (0.10, 0.55) | <0.001 |
| Sometimes | 0.12 (0.05, 0.31) | <0.001 |
| Frequently | 0.11 (0.04, 0.31) | <0.001 |
| Self evaluated job satisfaction (Ref: Dissatisfied) |  |  |
| Moderate | 0.62 (0.36, 1.10) | 0.092 |
| Satisfied | 0.21 (0.11, 0.39) | <0.001 |
| Age (Ref: ≤30) |  |  |
| 31-40 | 0.85 (0.52, 1.39) | 0.5 |
| 41-50 | 0.97 (0.54, 1.73) | >0.9 |
| >50 | 1.98 (0.93, 4.16) | 0.073 |
| Education (Ref: Junior /High /Technical school) |  |  |
| Vocational school | 1.20 (0.67, 2.16) | 0.5 |
| Bachelor degree or above | 1.05 (0.57, 1.97) | 0.9 |
| Sex (Ref: Male) |  |  |
| Female | 0.91 (0.58, 1.43) | 0.7 |
| Local residence (Ref: Local) |  |  |
| Migrant | 0.46 (0.02, 2.47) | 0.5 |
| Monthly income (Ref: < 3000 RMB) |  |  |
| 3000 - 5000 RMB | 0.90 (0.56, 1.46) | 0.7 |
| > 5000 RMB | 1.10 (0.60, 2.00) | 0.7 |
| Region (Ref: Eastern) |  |  |
| Central | 0.95 (0.51, 1.76) | 0.9 |
| Western | 1.40 (0.87, 2.28) | 0.2 |
| Northeastern | 1.52 (0.39, 4.76) | 0.5 |

**Table S14 Significant factors of depression among primary healthcare workers in rural area, including demographic characteristics.**

| Characteristic | OR (95% CI) | *P*-value |
| --- | --- | --- |
| Exercise status (Ref: Never) |  |  |
| Rarely | 0.49 (0.18, 1.35) | 0.2 |
| Occasionally | 0.47 (0.19, 1.19) | 0.10 |
| Sometimes | 0.24 (0.09, 0.66) | 0.004 |
| Frequently | 0.18 (0.06, 0.56) | 0.003 |
| Self evaluated job satisfaction (Ref: Dissatisfied) |  |  |
| Moderate | 0.66 (0.39, 1.15) | 0.14 |
| Satisfied | 0.23 (0.12, 0.43) | <0.001 |
| Self rated health status (Ref: Poor) |  |  |
| Fair | 0.31 (0.18, 0.55) | <0.001 |
| Good | 0.21 (0.12, 0.40) | <0.001 |
| Age (Ref: ≤30) |  |  |
| 31-40 | 0.82 (0.51, 1.31) | 0.4 |
| 41-50 | 0.91 (0.52, 1.58) | 0.7 |
| >50 | 1.64 (0.77, 3.43) | 0.2 |
| Education (Ref: Junior /High /Technical school) |  |  |
| Vocational school | 1.05 (0.60, 1.86) | 0.9 |
| Bachelor degree or above | 0.96 (0.53, 1.77) | >0.9 |
| Sex (Ref: Male) |  |  |
| Female | 1.00 (0.65, 1.55) | >0.9 |
| Local residence (Ref: Local) |  |  |
| Migrant | 1.00 (0.15, 3.95) | >0.9 |
| Monthly income (Ref: < 3000 RMB) |  |  |
| 3000 - 5000 RMB | 1.04 (0.65, 1.64) | 0.9 |
| > 5000 RMB | 1.13 (0.62, 2.01) | 0.7 |
| Region (Ref: Eastern) |  |  |
| Central | 1.12 (0.61, 2.03) | 0.7 |
| Western | 1.50 (0.94, 2.42) | 0.094 |
| Northeastern | 1.81 (0.50, 5.56) | 0.3 |

**Table S15 Significant factors of anxiety among primary healthcare workers in urban area, including demographic characteristics.**

| Characteristic | OR (95% CI) | P-value |
| --- | --- | --- |
| Living arrangement (Ref: Living alone) |  |  |
| Living with family members | 0.47 (0.32, 0.71) | <0.001 |
| Living with non-family members | 0.89 (0.50, 1.60) | 0.7 |
| Self evaluated job satisfaction (Ref: Dissatisfied) |  |  |
| Moderate | 0.24 (0.17, 0.33) | <0.001 |
| Satisfied | 0.10 (0.07, 0.14) | <0.001 |
| Self rated health status (Ref: Poor) |  |  |
| Fair | 0.30 (0.23, 0.40) | <0.001 |
| Good | 0.13 (0.08, 0.19) | <0.001 |
| Age (Ref: ≤30) |  |  |
| 31-40 | 0.83 (0.61, 1.14) | 0.3 |
| 41-50 | 0.68 (0.46, 1.00) | 0.051 |
| >50 | 0.94 (0.57, 1.53) | 0.8 |
| Education (Ref: Junior /High /Technical school) |  |  |
| Vocational school | 1.04 (0.61, 1.84) | 0.9 |
| Bachelor degree or above | 0.99 (0.58, 1.74) | >0.9 |
| Sex (Ref: Male) |  |  |
| Female | 0.98 (0.72, 1.34) | 0.9 |
| Local residence (Ref: Local) |  |  |
| Migrant | 0.95 (0.60, 1.47) | 0.8 |
| Monthly income (Ref: < 3000 RMB) |  |  |
| 3000 - 5000 RMB | 0.78 (0.57, 1.07) | 0.12 |
| > 5000 RMB | 0.77 (0.53, 1.12) | 0.2 |
| Region (Ref: Eastern) |  |  |
| Central | 0.82 (0.57, 1.17) | 0.3 |
| Western | 1.15 (0.86, 1.53) | 0.4 |
| Northeastern | 0.89 (0.53, 1.47) | 0.7 |

**Table S16 Significant factors of depression among primary healthcare workers in urban area, including demographic characteristics.**

| Characteristic | OR (95% CI) | P-value |
| --- | --- | --- |
| Exercise status (Ref: Never) |  |  |
| Rarely | 0.68 (0.42, 1.10) | 0.11 |
| Occasionally | 0.47 (0.30, 0.73) | <0.001 |
| Sometimes | 0.46 (0.28, 0.74) | 0.002 |
| Frequently | 0.43 (0.24, 0.75) | 0.003 |
| Living arrangement (Ref: Living alone) |  |  |
| Living with family members | 0.57 (0.38, 0.86) | 0.007 |
| Living with non-family members | 1.08 (0.61, 1.92) | 0.8 |
| Organizational support satisfaction (Ref: Dissatisfied) |  |  |
| Moderate | 0.40 (0.29, 0.57) | <0.001 |
| Satisfied | 0.27 (0.18, 0.40) | <0.001 |
| Self evaluated job satisfaction (Ref: Dissatisfied) |  |  |
| Moderate | 0.47 (0.33, 0.67) | <0.001 |
| Satisfied | 0.27 (0.17, 0.41) | <0.001 |
| Self rated health status (Ref: Poor) |  |  |
| Fair | 0.30 (0.23, 0.40) | <0.001 |
| Good | 0.13 (0.09, 0.19) | <0.001 |
| Age (Ref: ≤30) |  |  |
| 31-40 | 0.87 (0.65, 1.19) | 0.4 |
| 41-50 | 0.77 (0.53, 1.12) | 0.2 |
| >50 | 0.87 (0.53, 1.41) | 0.6 |
| Education (Ref: Junior /High /Technical school) |  |  |
| Vocational school | 1.23 (0.73, 2.12) | 0.5 |
| Bachelor degree or above | 1.04 (0.63, 1.80) | 0.9 |
| Sex (Ref: Male) |  |  |
| Female | 0.86 (0.64, 1.17) | 0.3 |
| Local residence (Ref: Local) |  |  |
| Migrant | 0.99 (0.64, 1.49) | >0.9 |
| Monthly income (Ref: < 3000 RMB) |  |  |
| 3000 - 5000 RMB | 0.78 (0.58, 1.07) | 0.12 |
| > 5000 RMB | 0.81 (0.57, 1.16) | 0.2 |
| Region (Ref: Eastern) |  |  |
| Central | 0.87 (0.62, 1.23) | 0.4 |
| Western | 1.33 (1.01, 1.75) | 0.044 |
| Northeastern | 1.30 (0.80, 2.04) | 0.3 |

**Table S17 Significant factors of anxiety among primary healthcare workers in rural areas clustered by hospital.**

| Characteristic | 95% CI | *P*-value |
| --- | --- | --- |
| Exercise status (Ref: Never) |  |  |
| Rarely | 0.28 (0.11–0.76) | 0.013 |
| Occasionally | 0.28 (0.12–0.67) | 0.004 |
| Sometimes | 0.16 (0.06–0.41) | <0.001 |
| Frequently | 0.15 (0.05–0.44) | <0.001 |
| Self evaluated job satisfaction (Ref: Dissatisfied) |  |  |
| Moderate | 0.60 (0.34–1.04) | 0.066 |
| Satisfied | 0.21 (0.11–0.39) | <0.001 |

Model fit: AUC = 0.73; marginal R² = 0.17; conditional R² = 0.13.

**Table S18 Significant factors of depression among primary healthcare workers in rural areas clustered by hospital.**

| Characteristic | 95% CI | *P*-value |
| --- | --- | --- |
| Exercise status (Ref: Never) |  |  |
| Rarely | 0.54 (0.20–1.47) | 0.226 |
| Occasionally | 0.52 (0.21–1.31) | 0.167 |
| Sometimes | 0.29 (0.11–0.77) | 0.013 |
| Frequently | 0.23 (0.07–0.68) | 0.009 |
| Self evaluated job satisfaction (Ref: Dissatisfied) |  |  |
| Moderate | 0.66 (0.39–1.13) | 0.131 |
| Satisfied | 0.25 (0.13–0.45) | <0.001 |
| Self rated health status (Ref: Poor) |  |  |
| Fair | 0.30 (0.18–0.53) | <0.001 |
| Good | 0.20 (0.11–0.38) | <0.001 |

Model fit: AUC = 0.75; marginal R² = 0.20; conditional R² = 0.22.

**Table S19 Significant factors of anxiety among primary healthcare workers in urban areas clustered by hospital.**

| Characteristic | 95% CI | *P*-value |
| --- | --- | --- |
| Living arrangement (Ref: Living alone) |  |  |
| Living with family members | 0.42 (0.28–0.62) | <0.001 |
| Living with non-family members | 0.91 (0.51–1.62) | 0.738 |
| Self evaluated job satisfaction (Ref: Dissatisfied) |  |  |
| Moderate | 0.24 (0.17–0.33) | <0.001 |
| Satisfied | 0.10 (0.07–0.15) | <0.001 |
| Self rated health status (Ref: Poor) |  |  |
| Fair | 0.31 (0.23–0.42) | <0.001 |
| Good | 0.13 (0.09–0.20) | <0.001 |

Model fit: AUC = 0.79; marginal R² = 0.25; conditional R² = 0.27.

**Table S20 Significant factors of depression among primary healthcare workers in urban areas clustered by hospital.**

| Characteristic | 95% CI | *P*-value |
| --- | --- | --- |
| Exercise status (Ref: Never) |  |  |
| Rarely | 0.66 (0.41–1.06) | 0.087 |
| Occasionally | 0.46 (0.29–0.71) | 0.001 |
| Sometimes | 0.44 (0.27–0.71) | 0.001 |
| Frequently | 0.42 (0.24–0.73) | 0.002 |
| Living arrangement (Ref: Living alone) |  |  |
| Living with family members | 0.51 (0.34–0.75) | 0.001 |
| Living with non-family members | 1.07 (0.61–1.89) | 0.805 |
| Organizational support satisfaction (Ref: Dissatisfied) |  |  |
| Moderate | 0.43 (0.30–0.60) | <0.001 |
| Satisfied | 0.28 (0.19–0.42) | <0.001 |
| Self evaluated job satisfaction (Ref: Dissatisfied) |  |  |
| Moderate | 0.47 (0.33–0.66) | <0.001 |
| Satisfied | 0.28 (0.18–0.42) | <0.001 |
| Self rated health status (Ref: Poor) |  |  |
| Fair | 0.31 (0.24–0.42) | <0.001 |
| Good | 0.14 (0.09–0.20) | <0.001 |

Model fit: AUC = 0.80; marginal R² = 0.28; conditional R² = 0.29.
