# Supplementary material for: Urban–rural differences in determinants of mental health among primary healthcare workers in China
Source: Epidemiol Psychiatr Sci. 2026 Jan 7;35:e4. doi: 10.1017/S2045796025100425 (PMC12816933; doi:10.1017/S2045796025100425)
Supplement: Huang et al. supplementary material 2 — Huang et al. supplementary material [file S2045796025100425sup002.doc]

STROBE Statement—Checklist of items that should be included in reports of ***cross-sectional studies***

|  | Item No | Recommendation | Page,  Line |
| --- | --- | --- | --- |
| **Title and abstract** | 1 | (*a*) Indicate the study’s design with a commonly used term in the title or the abstract | Page 1,  Line 1-11 |
|  |  | (*b*) Provide in the abstract an informative and balanced summary of what was done and what was found | Page 1-2,  Line 12-37 |
| Introduction | | |  |
| Background/rationale | 2 | Explain the scientific background and rationale for the investigation being reported | Page 2,  Line 40-53 |
| Objectives | 3 | State specific objectives, including any prespecified hypotheses | Page 2-4,  Line 54-92 |
| Methods | | |  |
| Study design | 4 | Present key elements of study design early in the paper | Page 4,  Line 96-97 |
| Setting | 5 | Describe the setting, locations, and relevant dates, including periods of recruitment, exposure, follow-up, and data collection | Page 4-5,  Line 98-124 |
| Participants | 6 | (*a*) Give the eligibility criteria, and the sources and methods of selection of participants | Page 5-6,  Line 125-133 |
| Variables | 7 | Clearly define all outcomes, exposures, predictors, potential confounders, and effect modifiers. Give diagnostic criteria, if applicable | Page 6,  Line 135-152 |
| Data sources/ measurement | 8* | For each variable of interest, give sources of data and details of methods of assessment (measurement). Describe comparability of assessment methods if there is more than one group | Page 6,  Line 135-152 |
| Bias | 9 | Describe any efforts to address potential sources of bias | None |
| Study size | 10 | Explain how the study size was arrived at | None |
| Quantitative variables | 11 | Explain how quantitative variables were handled in the analyses. If applicable, describe which groupings were chosen and why | Page 6,  Line 135-144 |
| Statistical methods | 12 | (*a*) Describe all statistical methods, including those used to control for confounding | Page 7,  Line 154-160 |
|  |  | (*b*) Describe any methods used to examine subgroups and interactions | None |
|  |  | (*c*) Explain how missing data were addressed | Page 8,  Line 187-188 |
|  |  | (*d*) If applicable, describe analytical methods taking account of sampling strategy | None |
|  |  | (*e*) Describe any sensitivity analyses | Page 8,  Line 185-187 |
| Results | | |  |
| Participants | 13* | (a) Report numbers of individuals at each stage of study—eg numbers potentially eligible, examined for eligibility, confirmed eligible, included in the study, completing follow-up, and analysed | Page 8,  Line 192-193 |
|  |  | (b) Give reasons for non-participation at each stage | None |
|  |  | (c) Consider use of a flow diagram | Figure S1 |
| Descriptive data | 14* | (a) Give characteristics of study participants (eg demographic, clinical, social) and information on exposures and potential confounders | Page 8-9,  Line 192-209 |
|  |  | (b) Indicate number of participants with missing data for each variable of interest | None |
| Outcome data | 15* | Report numbers of outcome events or summary measures | Page 9,  Line 210-223 |
| Main results | 16 | (*a*) Give unadjusted estimates and, if applicable, confounder-adjusted estimates and their precision (eg, 95% confidence interval). Make clear which confounders were adjusted for and why they were included | Page 10-11,  Line 228-276 |
|  |  | (*b*) Report category boundaries when continuous variables were categorized | Page 6,  Line 142-144 |
|  |  | (*c*) If relevant, consider translating estimates of relative risk into absolute risk for a meaningful time period | None |
| Other analyses | 17 | Report other analyses done—eg analyses of subgroups and interactions, and sensitivity analyses | Page 11,  Line 274-276 |
| Discussion | | |  |
| Key results | 18 | Summarise key results with reference to study objectives | Page 11,  Line 279-287 |
| Limitations | 19 | Discuss limitations of the study, taking into account sources of potential bias or imprecision. Discuss both direction and magnitude of any potential bias | Page 15,  Line 384-393 |
| Interpretation | 20 | Give a cautious overall interpretation of results considering objectives, limitations, multiplicity of analyses, results from similar studies, and other relevant evidence | Page 11-12,  Line 288-317;  Page 14,  Line 357-370 |
| Generalisability | 21 | Discuss the generalisability (external validity) of the study results | Page 12-14,  Line 318-356 |
| Other information | | |  |
| Funding | 22 | Give the source of funding and the role of the funders for the present study and, if applicable, for the original study on which the present article is based | Page 17,  Line 439-440 |

*Give information separately for exposed and unexposed groups.

**Note:** An Explanation and Elaboration article discusses each checklist item and gives methodological background and published examples of transparent reporting. The STROBE checklist is best used in conjunction with this article (freely available on the Web sites of PLoS Medicine at http://www.plosmedicine.org/, Annals of Internal Medicine at http://www.annals.org/, and Epidemiology at http://www.epidem.com/). Information on the STROBE Initiative is available at www.strobe-statement.org.
